# Patient perspectives and barriers to effective home-based care in lymphatic filariasis: A mixed methods study from Puducherry, India

**DOI:** 10.1371/journal.pntd.0013903

**Published:** 2026-01-13

**Authors:** Arya Rahul, Anoop C. Choolayil, Gnanasekaran Vijayalakshmi, Balakrishnan Vijayakumar, Dharani Govindasamy, Amala Ramasamy, Sadhasivam Anbusivam, Manju Rahi

**Affiliations:** ICMR-Vector Control Research Centre, Puducherry, India; The University of Sydney School of Veterinary Science, AUSTRALIA

## Abstract

Morbidity Management and Disability Prevention (MMDP) remain an under-addressed component of lymphatic filariasis elimination. Despite the simplicity of the hygiene-based regimen, adherence to these practices remains low due to multiple barriers, exacerbating chronic suffering. As the primary role remains vested in the patients, this study explores their perspectives and barriers to home-based care to inform strategies to improve outcomes. This study employed a mixed-methods approach using an explanatory sequential design. The quantitative phase involved a cross-sectional survey of 321 adult patients from Puducherry and adjacent areas of Tamil Nadu, utilising a validated questionnaire informed by the Self-Management Assessment Scale (SeMaS) framework. The qualitative phase comprised 12 in-depth interviews (IDIs) and four focus group discussions (FGDs), with participants evenly distributed based on gender and health-seeking behaviours, and the data were analyzed using deductive thematic analysis. Only 46.4% of participants regularly practised home-based limb care. Morbidity management scores were significantly higher among patients with an income (p = 0.005) and regular healthcare visits (p < 0.001), and lower among those with grade 4 lymphedema (p < 0.001). Participants under 60 years (p < 0.001), women (p = 0.015), and those with higher grades of lymphedema (p < 0.001) perceived a higher disease burden. Key barriers pertaining to home-based care were identified in social support (84.7%), perceived disease burden (33.0%), and mental health issues (15%). Qualitative findings highlighted the interactions of socioeconomic, structural, and cultural factors, indicating the key role of structural factors alongside individual-level determinants like locus of control, self-efficacy, and emotional well-being. The findings underscore the need for a multifaceted approach that transcends individual-level interventions to include systemic reforms, such as policy integration, capacity building, and community-driven support mechanisms. Addressing the barriers holistically and tailoring the solutions addressing diverse disadvantaged groups can enhance adherence to morbidity management practices.

## Author summary

Lymphatic filariasis is a neglected tropical disease that causes chronic suffering to the affected due to damage it inflicts on the lymphatic system, often resulting in lymphedema and associated disabilities. Managing lymphedema through home-based care is critical to improving patient outcomes, yet adherence to this simple regimen remains low. Given the global progress towards the elimination of lymphatic filariasis, it is important to improve outcomes for millions already affected. This study explores the barriers patients face in adhering to home-based limb care practices in Puducherry and Tamil Nadu, India. Using a mixed-methods approach, we conducted a survey with 321 patients and followed up with in-depth interviews and focus group discussions. We found that only 46.4% of participants consistently practised home-based care, with adherence significantly influenced by socioeconomic factors, healthcare access, and disease severity. Younger participants, women, and those with advanced lymphedema reported higher disease burdens. Key barriers included limited social support, high perceived disease burden, and mental health challenges. Our qualitative findings revealed that structural, socioeconomic, and cultural factors play critical roles in influencing adherence. The study highlights the need for policy integration, capacity building, and community-driven support mechanisms, alongside individual-level interventions to enhance morbidity management and disability prevention. These insights can guide targeted strategies to improve care for diverse disadvantaged groups affected by LF.

## Introduction

Lymphatic filariasis (LF) is a leading cause of permanent and long-term disability globally, with significant social and economic implications for affected communities [1]. The World Health Organization (WHO), in 1997, took a pivotal step by committing to the elimination of LF as a public health problem. This led to the launch of the Global Programme to Eliminate Lymphatic Filariasis (GPELF) in 2000, aimed at achieving LF elimination by 2020 through two main strategies: mass drug administration (MDA) to interrupt transmission and morbidity management and disability prevention (MMDP) to improve the quality of life for those already affected [2]. While substantial progress has been made under GPELF, with a notable 74% decline in LF infections worldwide (reducing cases to 51 million), many endemic regions have not yet met the 2020 elimination target guide [3]. This has prompted an extension of the goal to 2030, necessitating accelerated efforts to reach the target.

In India, the National Filaria Control Programme (NFCP), launched in 1955, prioritised disease prevention through MDA and vector control. Early assessments, however, revealed challenges such as community non-cooperation and vector resistance [4,5]. While subsequent policy revisions have improved elimination efforts, there remains a significant lag in implementing morbidity management and disability prevention (MMDP) activities, particularly in integrating home-based lymphedema management with primary healthcare services [6]. The latest efforts in the elimination of LF involve a renewed five-pronged strategy which includes biannual MDA, early diagnosis and treatment, integrated vector control, inter-sectoral convergence and leveraging digital platforms. These efforts reflect India’s commitment to achieving the elimination of LF by 2027, three years ahead of the global target set by the World Health Organization [7].

Lymphedema, a common chronic manifestation of filariasis, has no surgical or pharmaceutical cure, necessitating lifelong home-based self-care [2]. WHO guidelines recommend an Essential Package of Care focusing on a daily hygiene-based regimen, including washing and drying affected body parts to mitigate the risk of secondary infections [2]. Drawing from the WHO guidelines, the National Vector Borne Disease Control Programme (NVBDCP) recommends an essential package of care for the affected through health centres, including demonstration and training of self-care practices, treatment of acute episodes and hydrocele surgeries [8]. Basic management for lymphedema involves simple practices by the patient, including limb washing, skin care, foot care, limb elevation, exercises, wearing appropriate footwear, and wound care, along with management of Acute Dermato Lymphangio Adenitis (ADLA) episodes by a medical practitioner. Despite the simplicity of these measures, adherence remains low due to programmatic barriers, including insufficient funding, poor healthcare integration, lack of trained personnel, and limited awareness among patients and caregivers [9].

Despite the success of MDA in reducing transmission, globally the MMDP component remains largely neglected, leading to a significant gap in care for those suffering from chronic conditions like lymphedema [10]. As of 2023, India reported 621,178 lymphedema and 127,100 hydrocele cases [11]. Although the NVBDCP programme envisions providing essential care—including medical support and self-care training—the actual MMDP efforts are often confined to a one-time annual self-care demonstration and kit distribution during the MDA cycle, falling significantly short of the intended comprehensive care approach. Though the NVBDCP programme envisions essential care for the affected in terms of medical care and self-care training, the MMDP efforts are often limited to a one-time annual demonstration of self-care and distribution of MMDP kits during the annual MDA cycle, which significantly falls short of the envisioned comprehensive care plan. Self-care practices have proven to help improve health outcomes for lymphedema patients, but at the same time, the lack of focus on MMDP has been highlighted by several reports, emphasizing the need for comprehensive patient care to improve the quality of life of those affected and to serve as an indirect indicator of program success [12–14]. Moreover, the elimination efforts will leave behind a considerable population of patients requiring continued care, emphasizing the urgency of addressing these programmatic shortcomings [9].

The authors work closely with individuals affected by LF in Puducherry, an endemic region, through a specialised lymphatic filariasis clinic at ICMR-Vector Control Research Centre (VCRC), which is a WHO collaborating Centre for Research and Training in Lymphatic Filariasis. The clinic provides diagnosis, treatment, and morbidity management services to patients from Puducherry and neighbouring endemic states, including Tamil Nadu, Kerala and Andhra Pradesh. Our experience at the clinic shows poor adherence to the recommended hygiene regimen, even among patients who receive regular follow-up care at the clinic. Despite having access to clinical guidance, many patients face challenges in incorporating medical, behavioural, and emotional self-management practices into their daily routines. This study aimed to investigate the barriers to effective home-based care of filarial lymphedema from the patients’ perspective. By addressing these gaps, we seek to contribute evidence-based insights that can inform public health strategies and enhance the success of the LF elimination program.

## Method and materials

### Ethics statement

The study followed the National Ethical Guidelines for Biomedical and Health Research Involving Human Participants [15]. Ethical approval was granted by the Institutional Human Ethics Committee (IHEC/IRB No: IHEC-1122/N/J). Participation was voluntary, with the study’s purpose and objectives explained verbally and in a Participant Information Sheet (PIS). Before enlisting the participants, the investigators who were trained in qualitative research explained the study objective and procedures to the participants in their local language and willingness was enquired about. The participants were informed about their right to refuse an invitation or to withdraw from the study at any point in time. Participants were free to skip any uncomfortable questions or withdraw at any time. Written informed consent was obtained from all participants, and additional written consent was secured for audio-recording interviews. Confidentiality was ensured by anonymizing data using alphanumeric codes indicating participant ID, data collection mode, and gender. For example, a female participant in an IDI was coded as IDI_n_F, while a male participant in a focus group was coded as P(x)_FGD_M(n), where n represents the IDI/FGD number and x represents the assigned participant number in FGD.

This study employed a mixed-methods approach using an explanatory sequential design [16] with an initial quantitative phase, followed by an in-depth qualitative phase to explore and assess the perspectives of patients with filarial lymphedema on home-based care. The study was guided by the theoretical framework of Self-Management Screening (SeMaS), which is a framework for understanding patient-specific characteristics that could be barriers to efficacious self-management [17]. The initial quantitative inquiry, aided by the SeMaS instrument, helped understand the barriers to care spread across the six domains of the SeMaS framework, viz. Perceived Burden, Locus of Control, Self-Efficacy, Social Support, Coping, and Anxiety and Depression. Building on these insights, the subsequent qualitative phase delved deeper into each domain through Focus Group Discussions (FGDs) and In-Depth Interviews (IDIs), offering a nuanced understanding of the challenges faced by patients. The methodologies for both the quantitative and qualitative phases are comprehensively outlined in the following sections.

### Quantitative phase

The quantitative phase involved a cross-sectional survey of 321 patients aimed at assessing adherence to home-based care and identifying the barriers and facilitators influencing it. The study population consisted of adult filarial lymphedema patients (aged >18 years) in Pondicherry. The participants were categorised based on the WHO grading system for lymphedema [18]. However, as recommended by multiple studies, grades 4–7 were clubbed into one group (Grade 4: Severe) for the ease of analysis [19]. The study evaluated how the participants practised the WHO envisaged essential package of care (EPC) consisting of hygiene practices, skin and wound care, limb elevation, exercise and use of comfortable footwear. The appropriateness of footwear was assessed based on its comfort and suitability to the size and shape of the foot. Proper footwear should protect the feet from injury. Footwear that is too tight can cause injury and may precipitate acute attacks. Hence, footwear that causes feet to become hot, sweaty, or provides inadequate support is not advised [20]. In addition, two components, viz., massaging and compression bandaging, which have been reported to have a positive impact, were also evaluated.

The lymphatic filariasis clinic at ICMR-VCRC, Puducherry, currently has over 700 registered patients, although only about 200 are regular attendees. To capture a broad range of health-seeking behaviours affecting home-based care, the study included three distinct patient categories:

Category 1: Patients under regular follow-up at the filariasis clinic for at least one year.
Category 2: Patients registered with the clinic but who have not attended in the past six months.
Category 3: Patients who have not received treatment at any filariasis clinic for at least five years.

Participants in Categories 1 and 2 were selected from the lymphatic filariasis clinic operating under ICMR Vector Control Research Centre, Puducherry, India. Patients registered with this clinic were categorized based on the regularity of their attendance, and participants were chosen through simple random sampling using computer-generated random numbers. Patients with grade 1 lymphedema and/or cognitive disorders were excluded. If a selected patient did not meet the eligibility criteria, the next random number was considered. For Category 3, patients were identified through community health workers, and snowball sampling was employed to locate additional eligible participants. Recruitment continued until the desired sample size was achieved. Anticipating that at least 50% of the patients would be practising home-based care, the goal was to recruit a minimum of 100 patients per group. This would allow for results to be estimated with a relative precision of 20% and a 95% confidence interval, providing a robust sample for subgroup analysis. However, due to the uncertainty surrounding Category 3 patients, all identified patients during the study period were included in the sample.

#### Data collection.

Data for the quantitative phase was gathered using a questionnaire that was theoretically informed by the SeMaS framework [17]. The tool aimed at assessing the participants’ ability to manage their health independently, taking into account their skills, motivation, and support systems. The questionnaire consisted of three sections. The first section captured the sociodemographic information and clinical profile of the respondents. The sociodemographic aspect of the questionnaire used the modified Kuppuswamy scale for the year 2024 to capture the socioeconomic status of the respondents. The scale captures the education level and occupation of the head of the family, along with total per capita family income per month adjusted for the consumer price index to classify the participants into upper, upper middle, lower middle, upper lower and lower socioeconomic classes [21]. The second section consisted of a modified version of the SeMas tool, and the third section of the questionnaire captured the MMDP practices of the participants, rated on a five-point Likert scale, with higher scores indicating better MMDP practice. Questions on barriers to MMDP practices included predefined options refined through pilot surveys conducted among lymphatic filariasis patients attending the clinic, distinct from those enrolled in the study, along with an open-ended option to allow participants to elaborate on their perceived barriers. The questionnaire was in Tamil, the vernacular language of the respondents. The barriers listed were derived from the literature and expert opinions and then refined based on the preliminary findings from pilot interviews. The final version was designed to capture the barriers with a preset list, followed by an option to add any other barrier that the respondent raised. The morbidity management practices were assessed using a structured tool that captured multiple domains of home-based care, including limb washing, drying, nail care, interdigital space care, exercise, limb elevation, bandaging, limb massage, and footwear use. For each practice, both frequency (using a 5-point Likert scale ranging from Always to Never) and perceived barriers (knowledge gaps, physical constraints, pain, motivational issues, and social or occupational limitations) were documented. In addition, perceived benefits of limb care were captured using a 3-point Likert scale (Significant benefit, Moderate benefit, No benefit), along with self-reported clinical improvements.

The questionnaire underwent content validation, piloting, and reliability testing. Initially, a modified Delphi technique was employed with experts to validate the content, ensuring that the questionnaire comprehensively addressed all relevant domains and was well-structured. The questionnaire was piloted in two stages to assess its validity and reliability. In the first round, 10 participants completed the questionnaire and provided feedback, focusing on comprehension, clarity, appropriateness, and the adequacy of response options. Based on this feedback, the questions and response categories were refined. In the second round, the revised questionnaire was administered to an additional group of 20 participants to confirm comprehension and ensure the sufficiency of the response options. The reliability of the questionnaire was tested by measuring internal consistency using Cronbach’s alpha, with the final tool demonstrating a reliable alpha value of 0.761. The original English questionnaire was meticulously translated into Tamil (the vernacular language) with the help of a bilingual expert whose first language is Tamil and has good expertise in English and again the translated version was back-translated by another bilingual expert to ensure both linguistic and conceptual equivalence, thus minimizing the risk of misinterpretation.

Data collection was carried out by three trained investigators, ensuring consistency and reliability in the gathered information. Interview schedule for the quantitative phase was carried out by a senior nursing professional with over 30 years of experience in managing filariasis patients and conducting field surveys. The qualitative interviews were conducted by a medical doctor and a social scientist, trained in qualitative research methods and having experience working with lymphatic filariasis patients. At the conclusion of each interview, participants received counselling on morbidity management and disability prevention, thereby ensuring that the study provided direct benefits to the participants.

#### Data analysis.

Data were analyzed using STATA 14.2 [22]. Descriptive statistics were used to summarize the participants’ demographic and clinical characteristics, (MMDP) practices, and barriers encountered. The morbidity management practices were recorded on a 5-point Likert scale, and were scored with 2 points for adherence to recommended behaviors, 1 point for partially appropriate practices, and 0 points for no attempt. The SeMaS scoring system was applied to categorize participants based on the extent of barriers faced, using the original methodology [17]. The association of sociodemographic and clinical characteristics with morbidity management scores and perceived burden were analyzed using linear regression, adjusting for potential confounders. A p-value < 0.05 was considered statistically significant.

### Qualitative phase

In order to understand the factors that shape the “Self Management” practices of the respondents, an in-depth qualitative inquiry was employed among selected participants through IDIs and FGDs. This approach helped in exploring the lived experiences and contextual barriers in morbidity management [23]. The data thus generated was analysed with the SeMaS framework [17], using Atlas.ti software. Since the SeMaS framework provided a thematic structure, a deductive approach to data analysis was employed. Deductive thematic analysis, which begins with a predetermined set of themes or theoretical constructs, is particularly effective when aiming to test or apply existing frameworks to new contexts [24]. By focusing on specific categories of the SeMaS framework, i.e., Perceived Burden, Locus of Control, Self-Efficacy, Social Support, Coping, and Anxiety and Depression, the analysis directly addressed the theoretical lens, ensuring the findings are relevant to the study’s framework. Deductive coding streamlined the analysis, avoiding exploratory divergence and exclusively focusing on key barriers to self-management. This structured approach allowed for a focused exploration of barriers to self-management in chronic conditions, which are widely documented in the literature as critical factors in treatment adherence and quality of life outcomes [25]. Also, this facilitated triangulation, ensuring that the research actually answered the research question comprehensively [23,26].

#### Participant selection and data collection.

A set of respondents from the first phase, i.e., the quantitative phase, were purposively selected as the participants for the qualitative phase, to ensure a diverse representation of perspectives segregated by gender and treatment-seeking behaviour. The qualitative phase involved a combination of in-depth interviews and focus group discussions. Using multiple qualitative data collection methods ensured reaching robust conclusions about the research question through method triangulation [26–28]. Participants were informed about the study’s objectives and the voluntary nature of their involvement. Both the IDIs and FGDs took place at locations convenient for the participants and were conducted in Tamil, their native language. Separate interview guides, tailored for IDIs and FGDs, were developed based on existing literature, expert recommendations and findings from the quantitative phase [29–31]. While the core questions remained the same for IDIs and FGDs, the IDI interview guides included additional probe questions to elicit more nuanced responses from respondents, which sometimes cannot be elicited during FGDs due to their open nature.

The guide included probing questions to help keep participants focused while discussing their views. All IDIs and FGDs were audio-recorded after getting informed consent from the participants. Each IDI lasted between 40–60 minutes, while the FGDs ranged from 90 minutes to 2 hours. All IDIs and FGDs were audio-recorded with the participants’ consent. The content was then translated into English verbatim and reviewed by a bilingual expert to ensure the accuracy of the translation.

Two Separate FGDs were done each for male and female patients separated by their regularity in follow-up. The time and place of IDIs and FGDs were scheduled at the convenience of the participants. Each FGD lasted for one hour 30 minutes to 2 hours. Twelve IDIs with patients (under regular follow-up, irregular follow-up, and no follow-up) and four IDIs with caretakers equally distributed by gender were conducted. Each IDI lasted from 40 minutes to one hour (Table 1). Data saturation was ensured by including an adequate number of respondents across IDIs and FGDs, until new interviews or discussions yielded no additional insights beyond a certain threshold [32].

**Table 1:** List of FGDs and IDIs.

Focus Group Discussions		Participants	
FGD M1		Six male patients under regular follow-up(RF)	
FGD M2		Six male patients under irregular follow-up(IRF)	
FGD F1		Six female patients under regular follow-up(RF)	
FGD F2		Six female patients under irregular follow-up(IRF)	
**In-depth Interviews**			
IDI P1	Female patient under regular follow-up (F-RF)	IDI P2	Male patient under irregular follow-up (M-IRF)
IDI P3	Male patient under irregular follow-up (M- IRF)	IDI P4	Female patient not under follow-up (F-NF)
IDI P5	Female patient not under follow-up (F-NF)	IDI P6	Female patient under regular follow-up (F-RF)
IDI P7	Male patient under regular follow-up (M-RF)	IDI P8	Male patient not under follow-up (M- NF)
IDI P9	Female patient under irregular follow-up (F- IRF)	IDI P10	Male patient under regular follow-up (M-RF)
IDI P11	Female patient under irregular follow-up (F-IRF)	IDI P12	Male patient not under follow-up (M-NF)

#### Data analysis.

The transcripts of the IDIs and FGDs were analysed with the SeMaS framework [17], using Atlas.ti software. Since the SeMaS framework provided a thematic structure, a deductive approach to data analysis was employed. The content was analysed within the framework of the six domains of SeMaS framework viz. Perceived Burden, Locus of Control, Self-Efficacy, Social Support, Coping, and Anxiety and Depression. Each of these themes was probed in the data transcripts to understand factors contributing to poor MMDP practices. Relevant segments from the transcript were then coded by a researcher based on domains of the SeMaS framework. In order to ensure intercoder reliability, the coded data were then independently evaluated by two other researchers. Only the transcript segments corresponding to SeMaS domains with 100 per cent intercoder reliability were retained for triangulating with the quantitative data.

## Results

The study results present the perspectives of 321 patients with filarial lymphedema. The participants had a mean age of 59.9(±12.4) years. Category 1 (regular attendees) had a slightly higher proportion of male participants(51.5%), while Category 2 and 3 were dominated by females(62.4% and 58.0%, respectively). More than 92% of the participants belonged to the lower socioeconomic strata of the society according to the modified Kuppuswamy scale. Notably, 23% of the participants were illiterate, and another 205 had only primary school education. The sociodemographic details of the participants are enlisted in Table 2.

**Table 2:** Sociodemographic details of the study participants.

Variable	Patient category			Total n = 321 (%)
	Category 1(regular) n = 101 (%)	Category 2 (irregular) (n = 101) (%)	Category 3 (dropouts/ untreated) (n = 119) (%)	
**Gender**				
Female	49 (48.5)	63 (62.4)	69 (58.0)	181 (56.4)
Male	52 (51.5)	38 (37.6)	50 (42.0)	140 (43.6)
**Education**				
Graduate	2 (2.0)	9 (8.9)	4 (3.4)	15 (4.7)
High school/Diploma	28 (27.7)	18 (17.8)	15 (12.6)	61 (18.0)
Middle school	28 (27.7)	32 (31.7)	45 (37.8)	105 (32.7)
Primary school	21 (20.8)	22 (21.8)	23 (19.3)	66 (20.6)
Illiterate	22 (21.8)	20 (19.8)	32 (26.9)	74 (23.0)
**Occupation**				
Govt employee	8 (8.0)	7 (6.9)	8 (6.7)	23 (7.2)
Non-govt/self employed	17 (16.9)	17 (16.8)	13 (10.9)	47 (14.6)
Daily wage laborer	17 (16.8)	20 (19.8)	25 (21.0)	62 (19.3)
Unemployed	59 (58.4)	57 (56.4)	73 (61.3)	189 (58.9)
**Marital status**				
Married	76 (75.2)	70 (69.3)	82 (68.9)	228 (71.0)
Unmarried	4 (4.0)	6 (5.9)	7 (5.9)	17 (5.3)
Widow/ Widower/ Separated	21 (20.8)	25 (24.7)	30 (25.2)	76 (23.7)
Socio-economic status*				
Upper	–	–	–	–
Upper middle	3 (3.0%)	2 (2.0%)	1 (0.8%)	6 (1.9%)
Lower middle	8 (7.9%)	9 (8.9%)	2 (1.7%)	19 (5.9%)
Upper lower	66 (65.3%)	74 (73.3%)	93 (78.1%)	233 (72.6%)
Lower	24 (23.8%)	16 (15.8%)	23 (19.3%)	63 (19.6%)
**House type** ^**#**^				
Kucha	18 (17.8)	21 (20.8)	39 (32.8)	78 (17.8)
Pucca	83 (82.2)	80 (79.2)	80 (67.2)	243 (75.7)

* Socioeconomic status by Modified Kuppuswamy Scale 2024 [21]. House type classification: A kuccha house is a dwelling unit made from non-permanent materials such as mud, thatch, bamboo, or unburnt bricks. In contrast, a pucca house is constructed using durable materials like cement, concrete, burnt bricks, or stone [33].

Lymphedema was primarily unilateral across patient categories, though 22.7% had bilateral involvement, especially among category 2 and 3 patients. Of the participants, 38.9% had grade I or II lymphedema, 31.5% had grade III, and 29.6% had grade IV. Additionally, 29 male participants (20.7%) had concurrent hydrocele, with 4 cases presenting bilaterally. Among the hydrocoele patients, 6 had undergone surgery and 7 were not fit for surgery. More than half of the participants (51.4%) had associated intertrigo, 26.5% were diabetic, and 33.3% were hypertensive. Table 3 describes the clinical characteristics and comorbidities of the study participants.

**Table 3:** Clinical characteristics and comorbidities.

Variable	Patient’s clinic visits			Total n (%)
	Category 1(regular) n = 101 (%)	Category 2 (irregular) (n = 101) (%)	Category 3 (dropouts/untreated) (n = 119) (%)	
**Lymphedema laterality**				
Unilateral	84 (83.2)	75 (74.2)	89 (74.6)	248 (77.3)
Bilateral	17(16.8)	26 (25.8)	30 (25.4)	73 (22.7)
**Lymphedema grade** (highest grade in bilateral cases)				
**II**	33 (32.7)	39 (38.6)	53 (44.5)	125 (38.9)
**III**	29 (28.8)	35 (34.7)	37 (31.1)	101 (31.5)
**IV**	39 (38.6)	27 (26.7)	29 (24.4)	95 (29.6)
**Lymphedema durationMedian (Q1-Q3)**	25 (15–33)	20 (10–30)	25 (9–35)	20 (10–34)
**Hydrocele (in male patients)**	(n = 52)11 (21.1)	(n = 38)10 (26.3)	(n = 50)8 (16.0)	(n = 140)29 (20.7)
**Intertrigo**	57 (56.4)	47 (46.5)	61(51.2)	165 (51.4)
**Presence of comorbidities requiring long-term medication**	48 (47.5)	45 (44.5)	64(53.8)	157 (48.9)
**Hypertension**	34 (33.7)	28 (27.7)	45(37.8)	107 (33.3)
**Diabetes**	25 (24.8)	23 (22.8)	34(28.6)	85 (26.5)

### Home-based care of filarial lymphedema and the identified barriers

Self-care practices such as limb washing, drying, nail clipping, interdigital space care, limb massage, compression bandage application, and use of appropriate footwear varied significantly across patient categories, with regular patients demonstrating a higher adherence rate. Regular adherence to home-based limb care instructions was reported only by less than half (46.45%) of the study participants, and could reach only up to 78.2%, even among the regular clinic attendees. Knowledge gap (30.5%) was identified as the most important barrier to home-based care, followed by practical challenges (11.2%) and motivational barriers (8.7%). Among the regular clinic attendees, practical challenges, motivational barriers, and time constraints dominated.

**Fig 1:**
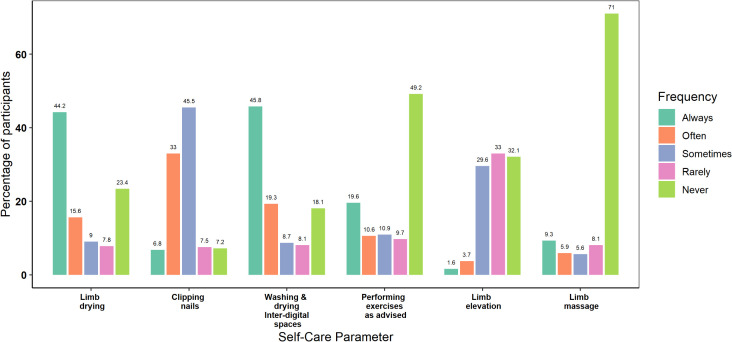
Adherence to self-care practices among the participants.

**Fig 2:**
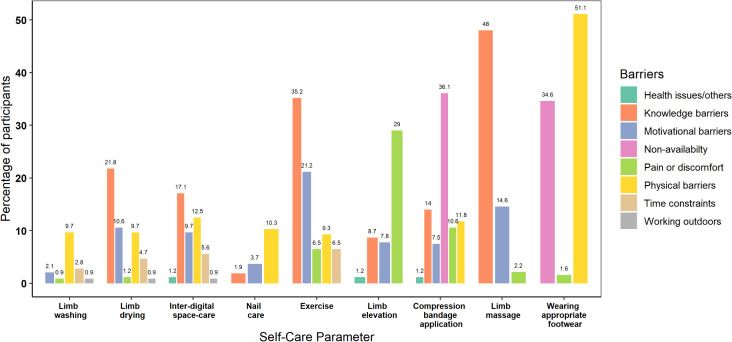
Perceived barriers to self-care practices among patients with filarial lymphedema.

Most (94.4%) of the participants adhered to instructions on limb washing, and physical difficulties prevailed to be the most important barrier across all the categories. Though the participants had a felt need for washing the limbs, the need for drying after washing was less perceived, with 100 (31.1%) participants never or rarely attempting to dry the limbs after washing. Figs 1 and 2 shows the adherence to various self-care practices among the participants and the major barriers perceived by them. While 21.8% of the participants were unaware of the need for drying the limbs, 10.6% did not feel motivated to make the effort despite the knowledge. A similar lacuna in knowledge (17.1%) and motivation (9.7) was observed in interdigital space care in addition to a major proportion reporting physical barriers (12.5%).

Even among the category 1 participants, only around half of them (52.5%) performed regular exercises as advised, with motivational (21.8%) and physical barriers (12.9%) along with pain-related factors (11.9%) predominating in this category of patients. Knowledge and motivational barriers were highlighted by category 2 and 3 patients. Several barriers were identified in the use of compression bandages including the application challenges, social stigma, bandage loosening, pain, and non-availability apart from motivational and knowledge barriers. Only 120 (44.8%) participants used appropriate footwear. Footwear fitting issues and financial constraints for purchasing custom footwear were identified as major challenges. A detailed compilation of home based MMDP practices and the perceived challenges among various categories of participants is given in S1 Table.

While 91.14% of the regular attendees perceived the benefit of home-based limb management, the proportion was only 52.4% of the total study participants. While 103 (32.1%) of the participants noted that home-based limb care prevented the progression of their lymphedema, 8.1% noted a reduction in edema and 11.8% noted a reduction in ADL attacks.

### Self-management screening

The results from the self-management screening questions highlighted variations in self-care capabilities, locus of control, self-efficacy, coping strategies, and emotional well-being among patients. Most patients, irrespective of their attendance category, reported a moderate disease burden (median score of 6).

The majority (94.6%) agreed that they were prepared to perform self-care of their limbs and knew the methods for limb washing and drying. However, around one-fifth of the participants (18.7%) lacked skills in the application of bandages. Patients’ locus of control revealed a shared belief across groups that personal efforts substantially influence health outcomes, with 83.2% agreeing with this statement. Notably, 56(17.5%) participants expressed their inability to perform the self-care activities as prescribed by the doctor. Social support emerged as a significant gap, with 84.7% of participants reporting substantial barriers to accessing it. The support networks comprised spouses (93 participants, 29%), children (73 participants, 22.7%), and others, including friends and relatives (34 participants, 10.6%).

Emotional coping strategies were the most commonly employed, with 20.5% of participants seeking comfort and understanding in difficult situations. Problem-solving behaviours were also prevalent, including efforts to improve their emotional state (14.3%) and deliberate actions to identify and resolve issues (13.8%). Avoidance behaviours, such as distraction or denial, were reported by 7.5% of participants.

Metrics on emotional well-being revealed that a considerable proportion of participants experienced anxiety and depressive symptoms. Among the participants, 112 (34.9%) frequently reported fear of embarrassment in social situations. Additionally, 31 (9.7%) often felt unable to experience happiness, with 27 (8.4%) perceiving everything as pointless. Alarmingly, 29 (9.0%) usually reported feelings of life not worth living.

**Fig 3:**
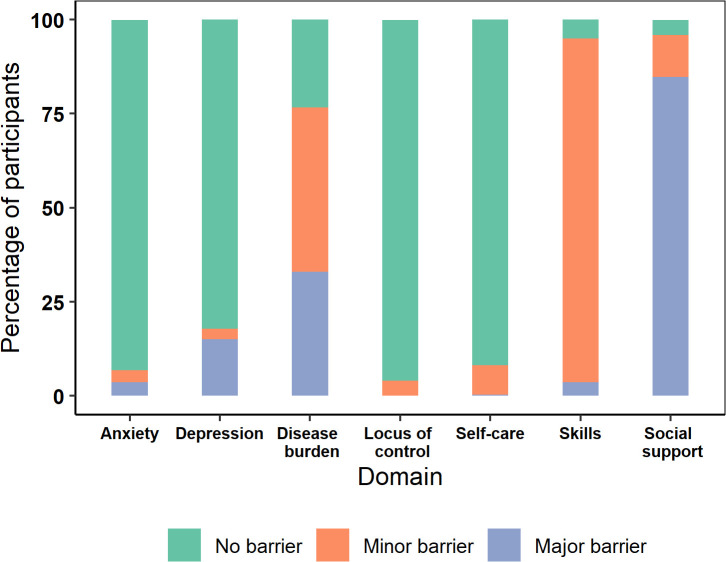
Categorisation of SeMaS scores of study participants.

On categorizing the data according to the experience of barriers, the majority of the patients faced barriers with social support, disease burden, and skills as shown in Fig 3. A notable proportion of 15.0% of participants had major barriers associated with mental health.

### Findings from the qualitative phase

**Fig 4:**
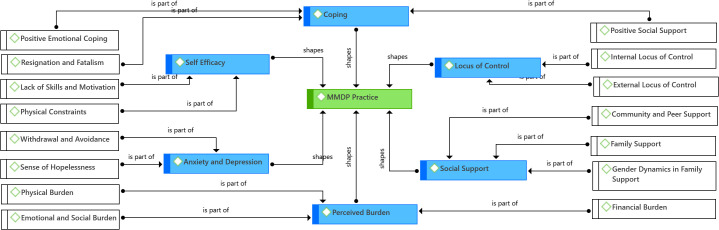
Factors shaping MMDP practice: SeMaS-informed qualitative findings.

To understand the factors that shape the self-management practices of the respondents, an in-depth qualitative inquiry was employed among selected participants through IDIs and FGDs. The findings from the qualitative phase are depicted in Fig 4 and thematically discussed in the following section.

#### Perceived burden.

In the context of lymphatic filariasis, perceived burden can be multifaceted, stemming from the physical manifestations of the disease and societal responses. The key domains of perceived burden affecting MMDP, as reported by the respondents, are listed below.

***Physical burden***: LF can induce significant physical challenges, such as pain, restricted movement and difficulty in performing day-to-day tasks. Physical burden manifested in two important forms among the participants viz. daily activities-related burden and job-related burden.

In terms of burden pertaining to daily activities, the majority of the patients thought that filariasis had affected their daily activities; though the magnitude varied. While some of the participants were able to do all their daily activities without external help, some participants were unable to accomplish their daily activities themselves. For instance, a female participant commented that even the use of the toilet posed challenges for her.

*This disease restricts many of my daily activities. I can’t bend or fold my legs; I am not able to sit and do activities like others. While washing clothes, I sit with my legs wide open and it is very difficult. I am not even able to use a toilet (Indian); I just sit on the floor.* (IDI_9_F)

Additionally, it was apparent from the interviews and FGDs that the disease had a major impact on the patient’s jobs and livelihoods. Patients narrated their experience of how they lost their jobs because of the disease, how their jobs exacerbated the risk for infections and ADLA attacks, and how their work affected their limb hygiene and home-based care activities.

*I work as a security personnel. My legs are swollen and I am unable to wear shoes. So, I’m working in a small place where they don’t demand to wear shoes. I wish to wear some good adjustable footwear, but couldn’t get one.* (P3_FGD_M2).

***Emotional and social burden***: The emotional burden associated with lymphatic filariasis significantly hinders individuals from engaging in essential MMDP practices. Emotional distress, including feelings of worthlessness, shame, and depression, reduces motivation and self-efficacy, making it difficult for individuals to adhere to self-care routines such as limb washing, bandaging, or exercising. Such pervasive feelings of inadequacy, often originating from social discrimination, may lead individuals to neglect self-care activities. Additionally, stigma and self-consciousness about the disease further discourage public engagement in MMDP, as seen in the following statement.

*I remove the bandage while going outside because I feel uneasy that I look disgusting to people.* (P1_FGD_F2).

Fear of social judgment can also exacerbate emotional distress, making individuals hesitant to seek help or consistently follow medical advice. For example, a participant commented:

*When I feel low, the weight of it all makes it easier to retreat into sleep than face the struggle of managing the disease.* (IDI_7_M).

***Financial burden:*** Financial strain is another critical barrier preventing individuals with LF from effectively practising MMDP.

*I tie the bandage myself, and though it gets dirty and loose, I can’t afford to replace it often.*(IDI_12_M)*.*

Financial instability can also lead to tension within families, further discouraging adherence to MMDP.

*We face a lot of financial struggles, and my husband resents me for seeking medical treatment, insisting that it’s pointless.* (IDI_9_F)*.*

Another participant stated that her poor economic background forced her to work to make ends meet, depriving her of the time to practice MMDP.

*I am doing household chores* [as a maid] *and earning 3000 rupees per month. I don’t have time to do all the self-care activities properly.* (IDI_6_F)

The participants also pointed out the lack of support from the state to cover their medical expenses.

*If the government supports us by giving some pension, it will be very helpful.* (P2_FGD_M2)

The perceived burden of lymphatic filariasis adversely affects MMDP practices. It not only reduces motivation and ability but also contributes to a sense of isolation and hopelessness among those affected. Addressing these burdens requires a comprehensive approach that combines physical rehabilitation, emotional counselling, community support, and financial assistance.

#### Locus of control.

Participants expressed varying degrees of perceived control over managing their lymphedema care. Some described taking active steps in their self-care routines despite challenges, while others felt helpless due to the progressive nature of the condition and external circumstances limiting their ability to manage symptoms effectively.

***Internal Locus of Control***: An internal locus of control is characterised by the belief that one’s own actions can positively influence health outcomes. Participants demonstrating this perspective often showed greater effort in self-care practices despite physical or emotional challenges. For instance, participant IDI_7_M commented:

*I know that if I wash my legs daily, I won’t get infections.* (IDI_7_M)*.*

Another participant stated how he understood that the disease, despite being incurable, can be controlled with MMDP.

*If we use medicines properly and maintain our legs clean, it can have a good impact.* (P3_FGD_F2)

***External Locus of Control***: An external locus of control reflects the belief that health outcomes are primarily determined by external forces, such as fate, the nature of the disease, or societal factors. This perspective often leads to feelings of helplessness and reduced adherence to MMDP practices. For instance, participant IDI_9_F commented:

*Whenever I overwork, especially with physical labour, my condition worsens—I often get a fever, and things quickly spiral downhill. If I rest and keep my leg clean, it stays manageable. But as a homemaker with many responsibilities, it’s not always possible, I am helpless.* (IDI_1_F).

Some respondents stated that they can do little about the disease progression and hence they are not in control.

*I have had this disease for many years. Even though I maintain my legs, the swelling keeps increasing. I don’t think anything can stop it.* (P1_FGD_M 2).

#### Self-efficacy.

Self-efficacy, in the context of this research, implies one’s confidence to manage LF and adhere to MMDP practices. Insights from IDIs and FGDs highlight the factors influencing self-efficacy, including physical challenges, time constraints, stigma, and availability of social support. Participants demonstrated varying degrees of self-efficacy in washing and drying their limbs. The participants had varying degrees of self efficacy shaped by multiple factors, of which the following were the predominant factors.

***Physical Constraints***: Physical ailments pose significant constraints to MMDP practices. The chronic and progressive nature of the disease results in swelling, pain, limited mobility, and recurring episodes of ADL, all of which hinder adherence to MMDP practices. Early adherence to MMDP can limit the progression to such stages, but later on, the physical challenges make practising MMDP difficult.

*I can’t do exercises because I’m unable to fold my legs. Sitting properly is also a challenge, and this leaves me feeling frustrated. Sometimes, I even have to stand while eating…* (IDI_7_M).

***Lack of Skills and Motivation***: Participants expressed difficulties in executing MMDP practices due to a lack of proper knowledge or confidence in their techniques. For instance, a male participant from the IDI commented that he was unable to execute bandaging properly.

*I tie the bandage, but it gets loose. I don’t know how to tie it properly so that it stays firm.* (IDI_7_M)

Lack of adequate skills or knowledge can lead to poor motivation among the patients, as they deem their efforts useless.

*If I don’t know the proper way to do things, it feels like whatever I do is useless.* (P2_FGD_F1)

Eventually it can lead to a sense of distrust in the MMDP practices and lead to poor motivation to practice MMDP.

*I don’t apply the bandage because it doesn’t seem to make a difference, and anyway, I feel too tired to do it regularly.* (P3_FGD_F2).

#### Social support.

Social support is a key facilitator of MMDP practices. The availability and quality of support from family and the broader community significantly influence adherence to MMDP practices. The narratives revealed diverse experiences, with the level of social support significantly facilitating or hindering adherence to MMDP practices.

***Family Support:*** Family members often play a pivotal role in assisting with daily self-care tasks. Participants shared instances where family support positively impacted their ability to manage their condition. For instance a participant narrated how her husband and daughter helped in better bandaging.

*If I apply the bandage by myself, it will loosen. So, I get help from my husband or daughter, and they tie it well.* (P5_FGD_F1)

However, the lack of family involvement can exacerbate feelings of isolation and hinder self-management.

*No one will help us. We have to do our work. If I ask for help from my husband, he will scold me.* (P1_FGD_ F1)

Family support, hence, not only alleviates the physical challenges of self-care but also enhances participants’ confidence in adhering to MMDP routines.

***Gender dynamics in family support:*** Gender dynamics often determine the nature of family support received by the participants to a great extent. Given the stereotypes associated with gender roles, female patients are often deprived of care and support from their spouse and immediate family, which can impact their MMDP practices. For instance, a female patient stated how she got little help from her husband in practising MMDP.

*If I ask for help from my husband, he will scold me and ask me to do it myself. It would have been better if I had a daughter.* (P1_FGD_F 1).

They remain responsible for the household chores even during acute illness episodes, which significantly affect the MMDP practices.

*Even when the wife is ill, she is responsible for completing all the household work.* (P3_FGD_F2)

In many societies, women are primarily responsible for domestic chores. These activities often involve exposure to water, prolonged standing, and other conditions not conducive to maintaining hygiene. Women’s roles as homemakers leave them with little time to focus on their own health needs.

*The doctor told me the intertrigo problem is due to moisture being retained between the toes. But with all my household activities I have to do, it is impossible to keep the feet always dry.* (P3_FGD_F1).

Conversely, some male participants noted their reliance on wives for support, though they acknowledged the strain this placed on their partners.

*My wife said, ‘Disease has caught us, we should not worry... We shall take care of it.’ She is my strength.* (P5_FGD_M 2).

***Community and peer support:*** While social support is often seen as a facilitator of health management, community and peer interactions can sometimes act as barriers to MMDP practices. Societal stigma, discriminatory attitudes, and lack of understanding discourage consistent self-care routines. For instance, a participant from the FGD commented that he avoided wearing bandages in public.

*If I wear a bandage in public, everyone looks at me and passes comments, so I prefer not to wear it when I go out.* (IDI_7_M*)*.

Such experiences create emotional barriers, discouraging individuals from practising visible MMDP activities. However, on the other hand, a supportive peer group can facilitate better emotional support and, thereby, better self-care. For instance, a participant commented that the support from her peer group helped her overcome her stress and made her life better.

*I am going to work to avoid my stress. I have a good workplace. People there are like a family, we share our thoughts, laugh together, have lunch together… They understand me. Everyone has their problems and we support each other. I have faced all the bitterness of my life with the support of good friends there*. (P6_FGD_F2).

#### Coping.

Coping strategies among individuals living with lymphatic filariasis significantly influence their MMDP practices. While positive strategies can enhance adherence, maladaptive approaches often act as barriers.

***Positive Emotional Coping:*** Many participants adopted positive emotional strategies to maintain resilience and focus on MMDP. This involved reframing their condition and drawing emotional strength from support systems. For instance, a participant from the FGD commented how accepting his condition helped him improve self-care.

*Now I understand that we have to take care of ourselves. We should not think about how others judge and make comments about us.* (P3_FGD_M1).

Another participant from the focus group discussion commented how staying positive helped him with the management of the disease.

*Initially, I was worried about how people would see me, but now I understand that this thought is killing me. It’s better not to dwell on it.* (P6_FGD_M2).

***Social Support as a Coping Mechanism:*** Social support plays an essential role in coping with the challenges of managing their condition and adhering to MMDP practices. The presence of supportive family members and community networks fosters psychological resilience, enabling individuals to manage the burden of the disease more effectively. For instance a participant commented how her granddaughters helped her cope with the disease.

*I spend my time with my four granddaughters. They make me happy and help me forget about the disease.* (P5_FGD_F2)

Another participant from the FGD narrated how support from the family is a key psychological component that improves resilience.

*My son massages my legs, and I am happy that my family doesn’t avoid me. It gives me the strength to manage this.* (P1_FGD_M2).

***Resignation and Fatalism:*** Resignation and fatalism can be pervasive and can hinder the motivation to engage in self-care practices, seek support, or pursue medical interventions, resulting in a further decline in health and well-being. For instance, some of the participants displayed a sense of resignation, which had a significant impact on their motivation to practice MMDP.

*I don’t apply the bandage because it doesn’t seem to make a difference, and anyway … I feel too tired to do it regularly.* (P3_FGD_F2).

Similarly, some participants resort to a sense of resignation, ignoring self-care. For instance, a participant from the FGD stated his efforts were futile, making him leave matters to fate.

*Even though I properly dry my legs, I am suffering from intertrigo … So I gave up on paying attention* (P6_FGD_M1).

Resignation and fatalism severely impact MMDP practices. These attitudes—often rooted in prolonged suffering, lack of social support, and a sense of helplessness—can lead to low motivation.

#### Anxiety and depression.

Anxiety and depression are common emotional responses among individuals with LF, significantly influencing their adherence to MMDP practices. Anxiety and depression due to LF is associated with multiple factors, of which some of the key aspects narrated by the participants of this study are listed below:

***Withdrawal and Avoidance***: Anxiety and shame about visible lymphedema often lead individuals to withdraw from social interactions. This withdrawal can hinder engagement in MMDP practices, such as attending clinics or wearing bandages. For instance, a participant from the FGD stated how anxiety about being discriminated against prevented him from travelling.

*Even a thief walks on the road without fear. But we are hiding our legs and walking like a thief due to fear of discrimination.* (P6_FGD_M 2).

Avoidance can have a dual impact, leading to weakened social support networks and reduced medical follow-ups, both of which adversely affect the patient’s well-being and care outcomes.

***Sense of Hopelessness***: A sense of hopelessness, driven by the chronic nature of the disease, undermines participants’ willingness to adhere to MMDP. This resignation often stems from the belief that their efforts are futile in halting disease progression. For instance, a male participant stated how he feels depressed about his condition leading to hopelessness and poor MMDP practices.

*I feel lonely and don’t think there is any point in washing or bandaging my legs daily. Nothing changes.* (IDI_8_M).

This leads to neglect of MMDP practices, accelerating disease progression and further reinforcing the cycle of hopelessness.

The analysis of the data from the qualitative phase following the SeMaS framework shows that MMDP practices among LF patients are highly shaped by Perceived Burden, Locus of Control, Self-Efficacy, Social Support, Coping, and Anxiety and Depression. By addressing these domains systematically, health interventions can empower patients to adhere to MMDP practices effectively.

An adjusted analysis of the factors associated with morbidity management and perceived burden scores is presented in Table 4. The morbidity management score was found to be significantly high in the employed participants including pensioners (β = 2.14; 95% CI: 0.66, 3.63; p = 0.005), low with advancing grade of lymphedema, Grade 4 (β = -2.92; 95% CI: -4.40, -1.44; p < 0.001). The perceived burden scores were significantly lower in the elderly(>60 years), β = -1.62; 95% CI: -2.18, -1.07; p < 0.001, and higher in females (β = 0.85; 95% CI: 0.16, 1.54; p = 0.015), patients with bilateral disease (β = 1.30; 95% CI: 0.65, 1.95; p < 0.001), Grade 3 lymphedema (β = 1.55; CI: 0.87-2.23; p < 0.001) and Grade 4 lymphedema (β = 2.99; 95% CI: 2.32, 3.65; p < 0.001).

**Table 4:** Factors associated with morbidity management score and perceived burden.

Variable	Morbidity management score^*^		Burden score^#^	
	β(95% CI)	P-value	β(95% CI)	P-value
**Age (>60)**	−1.15 (−2.35, 0.06)	0.063	−1.62 (−2.18, −1.07)	<0.001
**Female gender**	1.30 (−0.18, 2.79)	0.086	0.85 (0.16, 1.54)	0.015
**Occupation** (Employer/Pensioner)	2.14 (0.66, 3.63)	0.005	−0.32 (−1.01, 0.37)	–
**Grading of LF**				
Grade 3	−1.23 (−2.71, 0.24)	0.101	1.55 (0.87, 2.23)	<0.001
Grade 4	−2.92 (−4.40, −1.44)	<0.001	2.99 (2.32, 3.65)	<0.001
**Laterality (Bilateral)**	−0.18 (−1.58, 1.22)	−	1.30 (0.65, 1.95)	<0.001
**Patient group**				
New Patients	−10.06 (−11.46, −8.65)	<0.001	0.13 (−0.51, 0.77)	–
Irregular follow-up	−5.79 (−7.30, −4.29)	<0.001	−0.13 (−0.81, 0.54)	–

* R-square = 0.4521, p value of the model <0.001; #R-square = 0.3625, p value of the model <0.001.

## Discussion

The study highlights the gaps in home based-care of lymphatic filariasis that needs immediate public health attention. Patients with filarial lymphedema often face a double burden of physical and psychological distress, significantly affecting their quality of life [34]. The fact that LF affects the socially marginalised and the poor [35] signifies its importance as a public health problem, inducing the poverty health cycle [36]. Given home-based MMDP practice is the only available method for minimising the suffering caused by the disease [37], there needs to be more global attention to the barriers to MMDP. The implementation and practice of the “essential package of care” are not adequate to address the plight of the affected, mostly hailing from poor socio-economic conditions. Despite the substantial progress achieved under the GPELF, the neglect of MMDP continues to hinder the program’s ability to address the long-term implications of LF.

This study explored the perspectives of individuals suffering from filarial lymphedema on home-based care practices for filarial lymphedema and the factors that acted as barriers to its practice. The findings showed that the patients, irrespective of their follow-up patterns at a specialised clinic, reported challenges in practising MMDP, the major barriers identified being poor knowledge, lack of motivation and support, along with several physical and psychological barriers. A recent study from India has identified patient-related factors as a major barrier to MMDP, in addition to the inherent nature of the disease and health system-related factors [31]. They emphasised the need for improved service delivery and awareness creation among patients to achieve effective MMDP practices. Our study explores deeper into the patient-related factors and describes how individual and structural factors intersect, inducing poor adherence.

Anchored on the SeMaS framework, the study assessed variations in self-care capabilities, locus of control, self-efficacy, coping strategies, and emotional well-being among patients [17]. The cues from the quantitative phase were further investigated in-depth through IDIs and FGDs. The qualitative follow-up aimed at a nuanced understanding of each of the SeMaS domains in-depth. The findings from the qualitative phase unmasked the role of broader structural, social, and contextual factors that influence the ability of the patients to practice home-based limb care in addition to the affected individual’s motivation and effort. The following section triangulates the findings from quantitative and qualitative data to demonstrate this confluence of structural and individual factors shaping limb care practices.

The sociodemographic data of the quantitative part of the study showed some interesting patterns. For instance, the study had a higher proportion of female participants (56.4%). Notably, the skewness was contributed by a higher proportion of women in irregular clinic attendees/dropouts/untreated categories. This may be attributed to sociocultural barriers as women often face challenges like financial dependency, caregiving roles, and restricted mobility, limiting treatment adherence. Indications of these issues emerged in the qualitative interviews, where some female participants cited financial dependency and competing household responsibilities as factors behind low motivation in self-care. Studies have shown that stigma, lack of autonomy, and competing household responsibilities disproportionately affect women’s ability to sustain long-term care [38,39]. Additionally, the predominance of lower socioeconomic status (92%) highlights the socioeconomic barriers faced by individuals with filarial lymphedema, as highlighted by several studies [40,41].

The morbidity management score was significantly higher among participants with a steady income and pensioners showing that adherence to MMDP practices has an economic angle, with disproportionate adverse effects on the poorer sections. IDIs and FGDs revealed that participants with limited financial resources often faced severe constraints, compelling them to engage in physically demanding jobs that adversely affected their health. Job-related challenges hindered their ability to follow recommended limb care practices, creating a vicious cycle of poverty. The economic disadvantage forced them into manual labour and improper limb care, resulting in ADLA attacks, which led to wage loss and further disease progression, exacerbating both financial and health burdens. In contrast, individuals with better resources were able to practice MMDP more effectively, leading to better health outcomes. This underscores the critical role of social security measures and financial assistance in supporting these patients. Joy et al. identified significant gaps in the effective implementation of policies to include lymphedema patients under the Disability Act, highlighting disparities in enforcement, access to benefits, and the lack of an enabling ecosystem, compounded by administrative challenges, poor outreach, and inadequate intersectoral support. It is important to note that resource distribution is influenced not only by economic factors but also by structural and socio-cultural factors, such as gender roles. For instance, the perceived burden score was significantly higher among women, with many engaged in household chores or remunerative work due to traditional gender expectations, which impacted their self-care routines.

The significant proportion of patients with advanced lymphedema (61.1% with grade III or IV) underscores the chronicity and progression of the disease in resource-limited settings. Further, the morbidity management was found to be inversely associated with the grade of lymphedema. The prevalence of comorbidities such as diabetes (26.5%) and hypertension (33.3%) highlights the need for integrated care models, as previously noted by Mackenzie & Mante [10]. Yonzon et al. emphasized the socio-economic and cultural barriers faced by filariasis patients with hydrocoele, along with their limited knowledge, misconceptions and challenges they encounter [42]. The presence of concurrent hydrocele in male participants (20.7%) observed in the present study and the associated challenges—such as surgical ineligibility, recurrence, and patient reluctance despite efforts in line-listing and motivation for surgery—highlight the need for further exploration of specialized intervention strategies.

Poor adherence to home-based limb care instructions was influenced by multiple factors, including knowledge gaps, practical challenges, motivational barriers, lack of support, and low perceived value of the recommended practices. While adherence to limb washing indicated a basic level of awareness, significant gaps were observed in other components of the essential package of care, including drying the legs after washing, caring for interdigital spaces, limb elevation, exercise, wearing appropriate footwear, and accessing psychosocial support. Knowledge about these practices was notably poor among the dropouts and never-treated participants, emphasizing the importance of identification of these often-stigmatised patients and including them to the circle of essential lymphedema care [10]. This can be achieved by integrating lymphedema care into primary health systems and ensuring sustainable and accessible care for these patients [2]. Successful models from various settings have demonstrated the effectiveness of such integration, including community-based burden estimation strategies to achieve elimination targets and improve patient care [43,44].

In India, several initiatives focus on training medical and paramedical staff at the primary level to implement MMDP for lymphatic filariasis [8]. However, the attention given to line-listing patients and integrating their care into primary health systems remains limited compared to the emphasis on Mass Drug Administration for a long time [45]. In many settings, the burden of home-based limb care is largely left to patients and their families, underscoring the urgent need for systemic support. For instance, a study from south India revealed that only 70 per cent of the LF patients were aware of MMDP, among which only 48% practised it, indicating that the patients often bear the responsibility of MMDP without adequate systemic support [46]. Physical barriers such as pain and discomfort were frequently cited by the participants, particularly for activities like limb elevation, exercise, and massaging. Stigma further limited adherence to practices like bandaging, compounded by difficulties in application and maintenance. Financial constraints significantly hinder the use of appropriate footwear for lymphedema care, with fewer than half of participants using appropriate footwear. Unfortunately, little has changed compared to an Indian study conducted around 15 years back, reporting that most filarial lymphedema patients relied on ill-fitting or oversized footwear due to cost, poor quality, unsuitability for daily activities, and low-risk perception [47]. Recent studies from Africa also echo these findings, highlighting structural challenges rooted in poverty, alongside disease-specific issues like stigma and improper fit [48,49]. These findings underscore the need for tailored, context-specific support systems, such as community-based interventions, financial assistance for essential supplies, and motivational strategies like peer support and mobile health technologies, which have shown promise in addressing similar barriers [43,50–53].

Insights from the FGDs and IDIs highlight the interplay between disease burden and participants’ locus of control. While some patients believed positive actions could improve their health, others felt disease progression was beyond their control. Aligning with previous studies on locus of control and motivation [54], low internal locus of control often correlated with poor motivation and hopelessness, emphasizing the need for mental health support and social reinforcement to improve morbidity management practices. Additionally, many attributed this belief to life circumstances affecting lymphedema progression, despite their adherence to limb care practices. This underscores the need for interventions addressing structural factors beyond individual behaviours. The findings also revealed a link between self-efficacy, physical burden, and locus of control. Most participants were in late middle age or old age groups, with physical limitations that restricted them from the effective practice of home-based limb care, and many lacked the skills and motivation to practice it.

Although home-based care emphasizes family support, around 85% of participants in this study faced significant barriers in accessing such support. FGDs and IDIs revealed that social support, coping mechanisms, and mental health issues like anxiety and depression were closely interlinked. Participants with strong family and peer support exhibited positive coping strategies, while those lacking support often relied on maladaptive approaches such as resignation and fatalism. Gender norms further influenced family support, with most female participants reporting limited emotional and physical assistance due to entrenched stereotypes. This lack of support negatively impacted adherence to MMDP practices, underscoring the critical role of sociocultural factors in shaping self-care behaviours.

The significant mental health barriers (15%) identified in this study underscore the urgent need for policy-level interventions for integrated, comprehensive services. Lessons from Nigeria highlight strategies such as training MMDP providers in basic mental health care, routine mental health screening, and delivering mental health and MMDP services at the same facilities [55]. The interaction between gender and disability in terms of unmet mental health needs and access to care has also been highlighted [55]. Stigma remains a major obstacle, affecting both mental and physical health. Notably, many patients in this study reported limiting limb care activities due to stigma. The Stigma Elimination Programme (STEP) in Nepal demonstrated that improving self-care for leprosy patients helped reduce stigma, fostering dignity and empowerment [56,57].

The findings of this study emphasize the multifaceted barriers to adherence to home-based care of filarial lymphedema. The interplay of socioeconomic, structural, and cultural factors, alongside individual-level determinants such as locus of control, self-efficacy, and emotional well-being, highlights the complexity of addressing this issue. Hence, promoting MMDP practices requires interventions that address challenges at both individual and structural levels. This requires a shift from entrusting the burden of MMDP solely on the affected individuals to facilitating structural changes including systemic reforms, policy changes and community support.

We advocate for integrating home-based care into the primary healthcare system, supported by social and financial assistance and mental health interventions. Hands-on training using culturally sensitive communication strategies, support from field-level health workers, and access to essential resources such as medicines, reusable bandages, and customized footwear can improve self-efficacy and reduce the disease burden. An integrated approach to managing filariasis should prioritize stigma reduction, gender-sensitive interventions, and community involvement while addressing socioeconomic and mental health challenges. Addressing socioeconomic issues requires vocational rehabilitation, social assistance schemes, and sustainable funding models, including public-private partnerships. These schemes should not remain confined to policy documents but must be backed by strong system-level commitments to ensure effective implementation. Mental health monitoring and support for both patients and caretakers should be embedded in primary care, with specific strategies for patients lacking caretakers, such as integration into the palliative care system. Research on scalable, low-cost interventions and their long-term impact, as well as on locus of control and self-efficacy, will further enhance program inclusivity and effectiveness.

### Strengths and limitations

We used a mixed-methods approach to provide a comprehensive and in-depth understanding of the barriers and facilitators to home-based care among filarial lymphedema patients. The quantitative phase employed a well-defined sampling strategy and a validated tool, ensuring robust and reliable data collection. The qualitative phase enriched the findings by exploring the lived experiences of patients, guided by the SeMaS framework, which enabled a focused analysis of critical themes. However, some limitations warrant consideration. The self-reported data in the quantitative phase could be prone to recall or social desirability bias. Additionally, the dual role of investigators as both data collectors and counsellors might have influenced participant responses, particularly in reporting adherence. Despite these potential biases, adherence to limb care instructions was found to be generally poor among participants. The deductive analysis in the qualitative phase, while structured, may have restricted the emergence of novel themes outside the SeMaS framework. Furthermore, the context-specific nature of the study limits the generalizability of findings to other regions or populations. To address these challenges, we sought to capture diverse perspectives by recruiting three distinct categories of patients based on their healthcare-seeking behavior, a factor shown to directly influence morbidity management practices.

## Conclusion

This study underscores the critical gaps in home-based care for filarial lymphedema, highlighting the urgent need for comprehensive public health interventions. Adherence to home-based MMDP practices was found to be very low among the participants, with a poor perception of the benefits of these practices. The findings highlight the interplay of physical, socioeconomic, cultural, cognitive and psychological factors that influence adherence to these practices. Major barriers were identified in social support, and perceived disease burden with notable barriers in mental health. While personal motivation and self-care capabilities are essential, broader structural barriers, including gender norms, financial constraints, and limited social support, significantly hinder effective self-management. The findings underscore the need for a multifaceted approach that transcends individual-level interventions to include systemic reforms, such as policy integration, capacity building, and community-driven support mechanisms. Addressing these barriers holistically, and tailoring solutions to the diverse needs of the affected population, particularly women and socioeconomically disadvantaged groups, can enhance adherence to morbidity management practices. As the global goal of LF elimination nears, leaving many affected individuals behind, such measures are essential to bridge program gaps and ensure sustainable improvements in their quality of life. Collaborative efforts among healthcare providers, policymakers, and communities are crucial to achieving both LF elimination and comprehensive patient care.

## Supporting information

S1 Table Adherence to morbidity management practices among the participants and the identified barriers. (DOCX)
