# Supplementary material for: Patient perspectives and barriers to effective home-based care in lymphatic filariasis: A mixed methods study from Puducherry, India
Source: PLoS Negl Trop Dis. 2026 Jan 13;20(1):e0013903. doi: 10.1371/journal.pntd.0013903 (PMC12799011; doi:10.1371/journal.pntd.0013903)
Supplement: S1 Table — (DOCX) [file pntd.0013903.s001.docx]

**S1 Table.**

**Adherence to morbidity management practices among the participants and the identified barriers**

| Variable | Patient’s clinic visits | | | Total | P-value** |
| --- | --- | --- | --- | --- | --- |
|  | Category 1(regular)  n=101 (%) | Category 2 (irregular)  (n=101) (%) | Category 3 (dropouts/ untreated)  (n=119) (%) |  |  |
| Regular adherence to home-based limb care instructions | 79(78.2%) | 45(44.6%) | 25(21.0%) | 149(46.4%) | <0.001 |
| Major limitation faced for home-based care | | | | | |
| Knowledge gaps | 3(3.0%) | 21(20.8%) | 75(63.0%) | 98(30.5%) |  |
| Physical challenges | 16(15.8%) | 14(13.9%) | 6(5.0%) | 36(11.2%) |  |
| Time constraints | 7(6.9%) | 7(6.9%) | 5(4.2%) | 19(5.9%) |  |
| Motivational barriers | 7(6.9%) | 13(12.9%) | 8(6.7%) | 28(8.7%) |  |
| Lack of support | 5(5.0%) | - | 2(1.7%) | 7(2.2%) |  |
| Low perceived value | 4(4.0%) | 8(7.9%) | 1(0.8%) | 13(4.0%) |  |
| Limb washing | | | | | |
| Regular washing of the affected limb with soap and water | 100(99.0%) | 97(96.0%) | 106(89.1%) | 303(94.4%) | 0.043 |
| Frequency of limb washing | | | | | |
| Two or more times daily | 65(64.4%) | 58(59.2%) | 43(38.4%) | 166(52.4%) | 0.001 |
| Once daily | 36(35.6%) | 40(40.8%) | 69(61.6%) | 145(46.6%) |  |
| Barriers to limb washing | | | | | |
| Time constraints | 1(1.0%) | 4(4.0%) | 4(4.0%) | 9(2.8%) |  |
| Working outdoors | 1(1.0%) | 2(2.0%) | - | 3(0.9%) |  |
| Physical barriers | 12(11.9%) | 12(11.9%) | 7(5.9%) | 31(9.7%) |  |
| Motivational barriers | 2(2.0%) | - | 5(4.2%) | 7(2.1%) |  |
| Pain or discomfort | 2(2.0%) | 1(1.0%) | - | 3(0.9%) |  |
| Limb drying | | | | | |
| Frequency of drying limbs after washing | | | | | |
| Always | 49(48.5%) | 51(50.5%) | 42(35.3%) | 142(44.2%) | <0.001 |
| Often | 21(20.8%) | 15(14.8%) | 14(11.8%) | 50(15.6%) |  |
| Sometimes | 15(14.8%) | 7(6.9%) | 7(5.9%) | 29(9.0%) |  |
| Rarely | 5(5.0%) | 12(11.9%) | 8(6.7%) | 25(7.8%) |  |
| Never | 11(10.9%) | 16(15.8%) | 48(40.3%) | 75(23.4%) |  |
| Barriers in drying limbs | | | | | |
| Time constraints | 7(6.9%) | 5(5.0%) | 3(2.5%) | 15(4.7%) |  |
| Working outdoors | 1(1.0%) | 2(2.0%) | - | 3(0.9%) |  |
| Physical barriers | 16(15.8%) | 9(8.9%) | 6(5.0%) | 31(9.7%) |  |
| Knowledge barriers | 5(5.0%) | 15(14.9%) | 50(42.0%) | 70(21.8%) |  |
| Motivational barriers | 12(11.9%) | 12(11.9%) | 10(8.4%) | 34(10.6%) |  |
| Pain or discomfort | 1(1.0%) | 1(1.0%) | 2(1.7%) | 4(1.2%) |  |
| Nail care | | | | | |
| Frequency of clipping nails | | | | | |
| At least once in two weeks | 42(10.9%) | 37(4.9%) | 49(5.0%) | 128(6.8%) | 0.423 |
| Once in two weeks | 31(30.7%) | 32(31.7%) | 43(36.1%) | 106(33.0%) |  |
| Once in a month | 45(44.6%) | 45(44.6%) | 56(47.1%) | 146(45.5%) |  |
| Rarely | 9(8.9%) | 10(9.9%) | 5(4.2%) | 24(7.5%) |  |
| Never | 5(5.0%) | 9(8.9%) | 9(7.6%) | 23(7.2%) |  |
| Major difficulty in Nail care of your limbs | | | | | |
| Technical difficulty | 10(9.9%) | 8(7.9%) | 3(2.5%) | 21(6.5%) |  |
| Physical barriers | 10(9.9%) | 13(12.9%) | 10(8.4%) | 33(10.3%) |  |
| Knowledge barriers | - | - | 6(5.0%) | 6(1.9%) |  |
| Motivational barriers | 3(3.0%) | 3(3.0%) | 3(2.5%) | 12(3.7%) |  |
| Inter-digital space care | | | | | |
| Frequency of washing and drying inter-digital spaces | | | | | |
| Always | 50(49.5%) | 51(50.5%) | 46(38.7%) | 147(45.8%) | 0.002 |
| Often | 22(21.8%) | 17(16.8%) | 23(19.3%) | 62(19.3%) |  |
| Sometimes | 15(14.8%) | 7(6.9%) | 6(5.0%) | 28(8.7%) |  |
| Rarely | 7(6.9%) | 9(8.9%) | 10(8.4%) | 26(8.1%) |  |
| Never | 7(6.9%) | 17(16.8%) | 34(28.6%) | 58(18.1%) |  |
| Barriers to washing and drying inter-digital spaces | | | | | |
| Time constraints | 6(5.9%) | 8(7.9%) | 4(3.4%) | 18(5.6%) |  |
| Working outdoors | 3(3.0%) | - | - | 3(0.9%) |  |
| Physical barriers | 19(18.8%) | 13(12.9%) | 8(6.7%) | 40(12.5%) |  |
| Knowledge barriers | 4(4.0%) | 9(8.9%) | 42(35.3%) | 55(17.1%) |  |
| Motivational barriers | 11(10.9%) | 11(11.9%) | 8(6.7%) | 31(9.7%) |  |
| Exercise | | | | | |
| Frequency of performing the exercises advised | | | | | |
| Always | 32(31.7%) | 20(19.8%) | 11(9.2%) | 63(19.6%) | <0.001 |
| Often | 21(20.8%) | 12(11.9%) | 1(0.8%) | 34(10.6%) |  |
| Sometimes | 15(14.8%) | 10(9.9%) | 10(8.4%) | 35(10.9%) |  |
| Rarely | 12(11.9%) | 13(12.9%) | 6(5.0%) | 31(9.7%) |  |
| Never | 21(20.8%) | 46(45.5%) | 91(76.5%) | 158(49.2%) |  |
| Barriers in performing exercises | | | | | |
| Time constraints | 9(8.9%) | 10(9.9%) | 2(1.7%) | 21(6.5%) |  |
| Physical barriers | 13(12.9%) | 14(13.9%) | 3(2.5%) | 30(9.3%) |  |
| Pain | 12(11.9%) | 5(4.9%) | 4(3.4%) | 21(6.5%) |  |
| Knowledge barriers | 5(4.9%) | 24(23.8%) | 84(70.6%) | 113(35.2%) |  |
| Health issues | 1(1.0%) | 2(2.0%) | 1(0.8%) | 4(1.2%) |  |
| Motivational barriers | 22(21.8%) | 29(28.7%) | 17(14.3%) | 68(21.2%) |  |
| Limb elevation | | | | | |
| Practice of limb elevation | | | | | |
| >4 hours during the day | 3(3.0%) | 1(1.0%) | 1(0.8%) | 5(1.6%) | <0.001 |
| <4 hours during the day | 4(4.0%) | 3(3.0%) | 5(4.2%) | 15(3.7%) |  |
| During sleep only | 48(47.5%) | 20(19.8%) | 27(22.7%) | 95(29.6%) |  |
| Sometimes | 32(31.7%) | 43(42.6%) | 31(26.1%) | 106(33.0%) |  |
| Never | 14(13.9%) | 34(33.7%) | 55(46.2%) | 103(32.1%) |  |
| Barriers to practising limb elevation | | | | | |
| Pain/discomfort/sleep disturbance | 27(26.7%) | 31(30.7%) | 35(29.4%) | 93(29.0%) |  |
| Knowledge barriers | - | 2(1.0%) | 27(22.7%) | 29(8.7%) |  |
| Motivational barriers | 8(7.9%) | 12(11.9%) | 5(4.2%) | 25(7.8%) |  |
| Bandaging | | | | | |
| Frequency of compression bandage application | | | | | |
| Daily >5 hours | 20(19.8%) | 9(8.9%) | 7(5.9%) | 36(11.2%) |  |
|  |  |  |  |  |  |
| Daily <5 hours | 22(21.8%) | 5(5.0%) | 4(3.4%) | 31(9.7%) |  |
|  |  |  |  |  |  |
| Sometimes | 8(7.9%) | 3(3.0%) | 7(5.9%) | 18(5.6%) | <0.001 |
| Rarely | 11(10.9%) | 11(10 9%) | 12(10.1%) | 34(10.6%) |  |
| Never | 34(33.7%) | 70(69.3%) | 83(69.7%) | 187(58.3%) |  |
| Barriers to compression bandage application | | | | | |
| Social stigma | 11(10.9%) | 8(7.9%) | 7(5.9%) | 26(8.1%) |  |
| Occupational constraints including exposure to dirt/water | 14(4.0%) | 16(4.0%) | 15(2.5%) | 45(14.0%) |  |
| Application challenges | 19(18.8%) | 14(13.9%) | 10(8.4%) | 43(13.4%) |  |
| Bandage loosening/untying | 14(13.9%) | 5(5.0%) | 7(5.9%) | 26(8.1%) |  |
| Pain/ discomfort/restriction of movement | 16(15.8%) | 12(11.9%) | 6(5.0%) | 34(10.6%) |  |
| Non-availability | 6(5.9%) | 39(38.6%) | 51(42.9%) | 116(36.1%) |  |
| Motivational barriers | 7(6.9%) | 12(11.9%) | 5(4.2%) | 24(7.5%) |  |
| Knowledge barriers | 0 | 5(5.0%) | 40(33.6%) | 45(14.0%) |  |
| Presence of ulcer | 2(2.0%) | 1(1.0%) | 1(0.8%) | 4(1.2%) |  |
| Limb massaging | | | | | |
| Frequency of limb massage | | | | | |
| Always | 19(18.8%) | 9(8.9%) | 2(1.7%) | 30(9.3%) | <0.001 |
| Often | 12(11.9%) | 5(5.0%) | 2(1.7%) | 19(5.9%) |  |
| Sometimes | 7(6.9%) | 6(5.9%) | 5(4.2%) | 18(5.6%) |  |
| Rarely | 17(16.8%) | 8(7.9%) | 1(0.8%) | 26(8.1%) |  |
| Never | 46(45.5%) | 73(72.3%) | 109(91.6%) | 228(71.0%) |  |
| Barriers to practising limb massage | | | | | |
| Knowledge barriers | 15(14.9%) | 37(36.6%) | 102(85.7) | 154(48.0%) | - |
| Lack of assistance/caretaker | 10(9.9%) | 9(8.9%) | 2(1.7%) | 21(6.5%) |  |
| Time constraints | 13(12.9%) | 13(12.9%) | 2(1.7%) | 28(8.7%) |  |
| Motivational barriers | 19(18.8%) | 21(20.8%) | 7(5.9%) | 47(14.6%) |  |
| Pain | 6(5.9%) | 1(1.0%) | - | 7(2.2%) |  |
| Physical barriers | 21(20.8%) | 14(13.9%) | 3(2.5%) | 38(11.8%) |  |
| Appropriate footwear | | | | | |
| Footwear Usage | | | | | |
| Regular use of footwear | 93(92.1%) | 79(78.2%) | 96(80.7%) | 268(83.5%) | 0.017 |
| Use of appropriate footwear | 51(54.8%) | 40(50.6%) | 29(30.2%) | 120(44.8%) | 0.001 |
| Barriers to footwear usage | | | | | |
| Fitting issues | 35(34.7%) | 56(55.5%) | 73(61.3%) | 164(51.1%) |  |
| Lack of footwear-wearing habit | 3(3.0%) | 4(4.0%) | 5(4.2%) | 12(3.7%) |  |
| Financial constraints for custom footwear | 40(40.0%) | 35(34.6%) | 36(30.2%) | 111(34.6%) |  |
| Pain/Ulcer | 3(3.0%) | 1(1.0%) | 1(0.8%) | 5(1.6%) |  |
| Perceived benefit of instructions for limb management at home | | | | | |
| Significant benefit | 64(63.4%) | 32(31.7%) | 15(12.6%) | 111(34.6%) | <0.001 |
| Moderate benefit | 28(27.7%) | 22(21.8%) | 7(5.9%) | 57(17.7%) |  |
| No benefit | 9(8.9%) | 47(46.5%) | 97(81.5%) | 153(47.7%) |  |
| Reported improvements from adherence to limb management instructions | | | | | |
| Reduction in ADL attacks | 24(23.8%) | 12(11.9%) | 2(1.7%) | 38(11.8%) | - |
| Reduction in edema | 17(16.8%) | 5(5.0%) | 4(3.4%) | 26(8.1%) |  |
| No progression of edema | 60(59.4%) | 28(27.7%) | 15(12.6%) | 103(32.1%) |  |
| Reduced pain | 9(8.9%) | 8(7.9%) | 4(3.4%) | 21(6.5%) |  |
| Absence of intertrigo | 9(8.9%) | 2(2.0%) | - | 11(3.4%) |  |
| Healing of skin lesions | 10(9.9%) | 8(7.9%) | 1(0.8%) | 19(5.9%) |  |
| Improved ease of walking | 3(3.0%) | 2(2.0%) | - | 5(1.6%) |  |

**Chi-square test/Fisher’s exact test
